# Targeting DNA Methylation Depletes Uterine Leiomyoma Stem Cell–enriched Population by Stimulating Their Differentiation

**DOI:** 10.1210/endocr/bqaa143

**Published:** 2020-08-19

**Authors:** Shimeng Liu, Ping Yin, Jingting Xu, Ariel J Dotts, Stacy A Kujawa, John S Coon V, Hong Zhao, Ali Shilatifard, Yang Dai, Serdar E Bulun

**Affiliations:** 1 Division of Reproductive Science in Medicine, Department of Obstetrics and Gynecology, Feinberg School of Medicine, Northwestern University, Chicago, Illinois; 2 Department of Bioengineering, University of Illinois at Chicago, Chicago, Illinois; 3 Department of Biochemistry and Molecular Genetics, Northwestern University, Chicago, Illinois

**Keywords:** uterine leiomyoma, DNA methylation, stem cell differentiation

## Abstract

Uterine leiomyoma (LM) is the most common tumor in women and can cause severe morbidity. Leiomyoma growth requires the maintenance and proliferation of a stem cell population. Dysregulated deoxyribonucleic acid (DNA) methylation has been reported in LM, but its role in LM stem cell regulation remains unclear. Here, we fluorescence-activated cell sorting (FACS)-sorted cells from human LM tissues into 3 populations: LM stem cell–like cells (LSC, 5%), LM intermediate cells (LIC, 7%), and differentiated LM cells (LDC, 88%), and we analyzed the transcriptome and epigenetic landscape of LM cells at different differentiation stages. Leiomyoma stem cell–like cells harbored a unique methylome, with 8862 differentially methylated regions compared to LIC and 9444 compared to LDC, most of which were hypermethylated. Consistent with global hypermethylation, transcript levels of TET1 and TET3 methylcytosine dioxygenases were lower in LSC. Integrative analyses revealed an inverse relationship between methylation and gene expression changes during LSC differentiation. In LSC, hypermethylation suppressed the genes important for myometrium- and LM-associated functions, including muscle contraction and hormone action, to maintain stemness. The hypomethylating drug, 5′-Aza, stimulated LSC differentiation, depleting the stem cell population and inhibiting tumor initiation. Our data suggest that DNA methylation maintains the pool of LSC, which is critical for the regeneration of LM tumors.

Uterine leiomyoma (LM, or fibroid) is the most common tumor in women, affecting approximately 80% of women worldwide (1, 2). It affects African American women disproportionately, beginning at an earlier age and with a higher prevalence (1, 3). Although benign in origin, LM can cause significant morbidity, including excessive uterine bleeding, recurrent pregnancy loss, and pelvic pain; these symptoms may also mimic or mask malignant tumors (2). More than 200 000 surgeries are performed in the Unites States each year to remove LM, at an estimated cost of $5.9–34.4 billion (4, 5). Despite their prevalence and impact on women’s health, no new medical treatments have been approved in the United States for LM since the 1990s (6).

Stem cells are critical for normal tissue and disease development. Several studies have demonstrated the presence of stem cells in LM, which are indispensable for proliferation, regeneration, and tumor growth (7–9). Leiomyoma stem cells are deficient in estrogen and progesterone receptors, which may explain the frequent regrowth of LM that is observed after the discontinuation of hormone-based therapies (10). Using 2 cell surface markers, CD34 and CD49b, we identified 3 molecularly and functionally distinct cell populations in LM tissues, with a possible hierarchical differentiation order: LM stem cell–like cells ([LSC] CD34+/CD49b+, 5%), LM intermediate cells ([LIC] CD34+/CD49b-, 7%), and terminally differentiated LM cells ([LDC] CD34-/CD49b-, 88%) (10, 11). Microarray-based gene expression profiling demonstrated that the LSC population has a distinct transcriptome from LIC and LDC but how LSC maintain their unique gene expression profile and stemness remains unclear (11).

Epigenetic alterations, such as deoxyribonucleic acid (DNA) methylation, play crucial roles in stem cell regulation and disease progression (12, 13). Within cancer stem cells, the genes required for differentiation are suppressed by DNA methylation, leading to abnormal clonal expansion (14–17). In several cancer types, researchers have found that DNA-hypomethylating agents can “reset” cancer stem-like cells towards a differentiation phenotype, thereby resensitizing them to chemotherapy (17). Previously, it was demonstrated that, compared with myometrium, LM contain a dysregulated DNA methylation landscape, with hypermethylation identified around the promoter regions of tumor suppressors such as KLF11, DLEC1, and KRT19 (18, 19). It remains unclear how DNA methylation affects LSC differentiation and tumorigenesis.

In this study, we characterized the global transcriptomes and methylomes of each LM cell population and evaluated the role of DNA methylation during the LSC differentiation process. We hypothesized that DNA methylation “locks” genes critical for differentiation in a suppressed state, which maintains LSC function, and how removing this epigenetic suppression would drive LSC differentiation, resulting in the depletion of the LSC population.

## Materials and Methods

### Tissue collection

Northwestern University’s Institutional Review Board approved the use of human tissue. Leiomyoma tissues were obtained from premenopausal women undergoing either myomectomy or hysterectomy (age 38 ± 9 years, range 29–47 years). Informed consent was obtained from all participants prior to tissue collection. Patients receiving hormone treatment within 6 months prior to surgery were excluded. Information of race/ethnicity and MED12 mutation status for patient samples used for next generation sequencing (RNA-Seq and MethyCap-Seq) are listed in Table 1. Tissues were dissociated and cells were isolated, as previously described (10).

**Table 1.** Ethnicity and MED12 Mutation Status of Patient Samples

Sample	Race/Ethnicity	MED12 status	HMGA2 OE
LSC, LIC, and LDC MethylCap-Seq PT1	African American	MED12 G44S	No
LSC, LIC, and LDC MethylCap-Seq PT2	African American	MED12 G44S	No
LSC, LIC, and LDC MethylCap-Seq PT3	African American	WT	No
LSC, LIC, and LDC RNA-Seq PT1	Caucasian	WT	No
LSC, LIC, and LDC RNA-Seq PT2	Declined to answer	WT	No
LSC, LIC, and LDC RNA-Seq PT3	African American	WT	No
LSC, LIC, and LDC RNA-Seq PT4	African American	G44V	No
5’-Aza or Veh-treated LM RNA-Seq PT1	Caucasian	WT (after culture)	No
5’-Aza or Veh-treated LM RNA-Seq PT2	Caucasian	WT (after culture)	No
5’-Aza or Veh-treated LM RNA-Seq PT3	African American	WT (after culture)	No

Abbreviations: G44S, glycine to Serine mutation; HMGA2, high Mobility Group AT-Hook 2; LDC, leiomyoma differentiated cells; LIC, leiomyoma intermediated cells; LSC, leiomyoma stem cells; LM, Leiomyomas; MED12, Mediator complex subunit 12; OE, Overexpression; PT, patient; WT, wildtype.

### Primary cell and tissue explant culture

Primary LM cell and ex vivo explant culture of LM tissue were performed, as previously described (20). Briefly, primary LM cells were cultured in 6-well or 100-mm cell culture plates with DMEM/F12 (Thermo Fisher Scientific, Waltham, Massachusetts) containing 10% fetal bovine serum and 1% antibiotic/antimycotic in a humidified atmosphere, with 5% CO_2_ at 37°C. Cells were treated with vehicle 5′-Aza (25 nM, 50 nM, or 100 nM; A3656, Sigma-Aldrich, St. Louis, Missouri) or RU486 (10^–6^ M) for 6 days, with the culture medium refreshed every day. To elucidate the role of 5′-Aza and RU486 in LSC function, fresh tissues were minced to small pieces (1 mm^3^) and treated with vehicle 5′-Aza (100 nM) or RU486 treatment (10^–6^ M) for 48 hours. Then, single cells were dissociated from the treated explants and analyzed using flow cytometry based on the expression of CD34 and CD49b, as previously described (10).

### Three-dimensional spheroid culture

Each freshly Fluorescence-activated cell sorting (FACS)-sorted population of LM cells was cultured in mesenchymal stem cell growth medium (PT-3238, Lonza, Basel, Switzerland) in low-attachment 96-well plates (07-201-680, Fisher Scientific, Hampton, New Hampshire) to maintain stem cell characteristics. Cells were recovered in mesenchymal stem cell growth medium for 3 days, followed by treatment with 5′-Aza (100 nM) or vehicle for 96 hours.

### Antibody and primer information

All antibodies and primers used in this manuscript are listed in Table 2.

**Table 2.** List of Antibodies and Primers

Antibodies	Company	Cat. Number	Usage
CD45	BD Biosciences	564105	FACS
CD34	BD Biosciences	555824	FACS
CD49b	BD Biosciences	555498	FACS
H3K4me3	Millipore	07-473	ChIP-Seq
H3K27Ac	Active Motif	39133	ChIP-Seq
H3	Active Motif	39763	ChIP-Seq
**Expression primers**	**Company**	**Assay ID**	**Probe**
TET1	Integrated DNA Technologies	Hs.PT.58.27802060	Taqman
TET3	Integrated DNA Technologies	Hs.PT.58.4763348	Taqman
ESR1	Life Technologies	Hs00174860_m1	Taqman
TIMP3	Integrated DNA Technologies	Hs.PT.58.1756331	SYBR
ROR2	Integrated DNA Technologies	Hs.PT.58.22908006	SYBR
MYH11	Integrated DNA Technologies	Hs.PT.58.2909933	SYBR
TBP	Life Technologies	Hs00427620_m1	Taqman

Abbreviation: FACS, fluorescence-activated cell sorting.

### Antibody-based cell sorting

CD34^+^/CD49b^+^, CD34^+^/CD49b^-^, and CD34^-^/CD49b^-^ LM cells were FACS-sorted, as previously described (10, 20).

### Ribonucleic acid isolation, real-time quantitative polymerase chain reaction, and RNA-Seq

Total ribonucleic acid (RNA) was isolated using the Qiagen Allprep RNA/DNA mini kit (80204, Qiagen) or Qiagen RNeasy RNA micro kit (74004, Qiagen, Hilden, Germany). Complementary deoxyribonucleic acid was synthesized using qScript cDNA SuperMix (total RNA >100 ng; 95048–100, VWR International, Radnor, Pennsylvania) or SuperScript VILO Master Mix (total RNA <100 ng; 11754050, Thermo Fisher Scientific, Waltham, Massachusetts). Messenger RNA (mRNA) levels were quantified using real-time quantitative polymerase chain reaction (qPCR) normalized to TATA-BOX Binding Protein (TBP), as previously described (21). Total RNA quality was examined using the Bioanalyzer RNA Pico assay (Agilent, Santa Clara, California). RNA-Seq libraries were prepared using the NEBNext Ultra II RNA Library Prep with Sample Purification Beads (7775, NEB, Ipswich, Massachusetts).

### MethylCap-Seq

Genomic DNA was extracted from sorted LM cells using the Qiagen Allprep RNA/DNA mini kit (80204, Qiagen, Hilden, Germany) and was fragmented to 300–500 bp using Covaris M220 (Covaris, Woburn, Massachusetts). Methylated DNA fragments were captured using the MethylCap Kit (C02020010, Diagenode, Sparta, New Jersey) following the manufacturer’s protocol. Briefly, 500 ng of fragmented genomic DNA was incubated with H6-GST-MBD fusion proteins that bind methylated cytosines. Previous studies have shown that a single, fully methylated CpG is sufficient for the interaction between the H6-GST-MBD fusion protein and methylated DNA (22). The protein-DNA complex was then precipitated with antibody-conjugated beads specific to the protein tag. The immunoprecipitated DNA fragments were purified and subjected to library construction and next-generation sequencing, as described below. Deep sequencing provides greater genome coverage, representing the majority of MBD-bound methylated DNA.

### Native ChIP-Seq for histone modification

We followed the protocol published by Peter van Galen et al for native ChIP-Seq on histone modifications using low cell numbers (23). All sequencing barcodes used in this study were provided in the original publication (23).

### Next-generation sequencing

Next-generation sequencing libraries for MethylCap-Seq were prepared using the KAPA Hyper Prep Kit (KK8502, KAPA Biosystems) and KAPA Single-Indexed Adapter Kit (KK8710, KAPA Biosystems, Wilmington, Massachusetts). The libraries were sequenced at the Northwestern University NUSeq Core Facility using the NextSeq 500 system (Illumina, San Diego, California), with 20–40 million reads per sample. Sequencing methods were 75 bp or 50 bp single-end for RNA-Seq and 75 bp paired-end for MethylCap-Seq and histone modification ChIP-Seq.

### Bioinformatics analysis

Sequences were aligned to the hg19 reference genome using TopHat for RNA-Seq, Bowtie2 for MethylCap-Seq, and Burrows-Wheeler Aligner for histone modification ChIP-Seq (24–26). Differential gene expression from RNA-Seq was detected using DESeq2 and following the cutoff of False Discovery Rate (FDR)-adjusted *P *< 0.05. We performed the following MethylCap-Seq analyses using Homer software: peak callings (-histone for MethylCap-Seq), differential methylation analyses (getDifferentialPeaksReplicates.pl, FDR-adjusted *P *< 0.05, fold-change >2), and peak annotations (annotatePeaks.pl) (27). Pathway enrichment analysis was performed using Metacore V6.34 (Thomson Reuters, Toronto, Canada) and Gene Enrichment Analysis (GSEA, University of California San Diego [San Diego, California] and Broad Institute [Cambridge, Massachusetts]) V4.0.1 (28, 29). BAM files from replicates were merged by samtools before NGS.plot and UCSC Genome Browser, University of California Santa Cruz (Santa Cruz, California) visualizations (30). Sequencing tracks were visualized using the UCSC Genome Browser. Visualization of DNA methylation and histone modification average levels at specified regions (gene body regions and differentially methylated regions) were performed using NGS.plot (31). Methylation levels at polycomb target regions were quantified on BAM files using the normalized RPKM value (log2(RPKM_IP_ + 1) – log2(RPKM_Input_ + 1)).

### Colony formation assay

Primary LM cells were treated with vehicle (DMSO; Sigma-Aldrich, St. Louis, Missouri), 5′-Aza, or RU486 for 6 days, as described under primary cell and tissue explant culture. After treatment, 500 viable cells were seeded in each well of 12-well plates and cultured for 21 days without any treatment. Plates were then washed with 2 mL PBS, fixed with 3 mL 10% formalin (252549, Sigma) for 15 minutes, and stained with crystal violet for 5 minutes (0.025% w/v; HT90132, Sigma-Aldrich, St. Louis, Missouri). The number of colonies was counted in each well, excluding small colonies (<50 cells) (32).

### Animal studies

Northwestern University’s Animal Care and Use Committee approved all procedures involving animals. For each xenograft, 10^6^ live cells were grafted underneath the kidney capsules of ovariectomized nonobese diabetic-scid IL2Rgnull mouse hosts (NSG, Jackson Laboratory, Bar Harbor, Maine), as previously described (20). To test whether 5′-Aza affects the tumor-initiation capacity of primary LM cells, cells were treated with vehicle (DMSO), 5′-Aza (100 nM), or RU486 (10^–6^ M) for 6 days prior to xenograft. Mice were euthanized 4 weeks postsurgery. Images of regenerated tumors on the kidney surface were taken from the x-, y-, and z-axes using a dissecting microscope connected to a computer with Leica Application Suite, version 3.8 software (Leica Microsystems Inc, Wetzlar, Germany). Tumors were measured by 2 individuals who were blinded to the treatment group allocation; the average measurement taken by the 2 individuals was used for data plotting. Tumor volume was quantified using the following formula: volume (mm^3^) = 0.52 (derived from π/6) * length * width * height (mm^3^) (33).

### Statistical analysis

All statistical analyses were performed using GraphPad Prism 8 (GraphPad Inc., San Diego, California) and R (3.6.0); detailed statistical test descriptions are reported in each figure legend. No sample was excluded during the analysis. Values were considered statistically significant when *P *< 0.05. All experiments were repeated with samples from at least 3 patients, with the patient number (n) noted in the figure legends. Data points in the bar plots represent biological replicates from different patients or mice, and the error bars represent Standard error of the mean.

## Results

### The LSC population has a unique DNA methylation landscape

**Figure 1.**
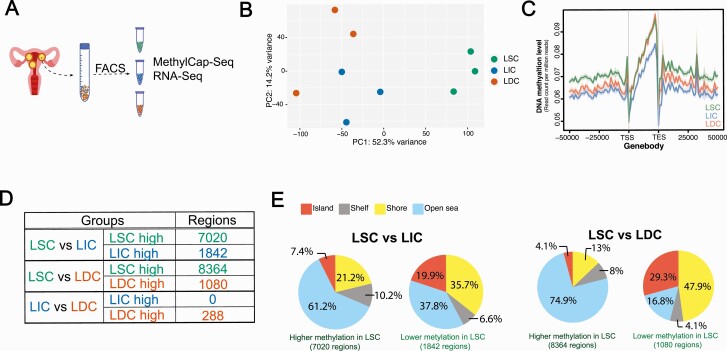
The LSC population harbors a unique DNA methylation landscape. **A**: The workflow of LM cell population characterization. **B**: PCA plot showing the DNA methylation landscape of each LM population (n = 3 patients, top 10 000 methylated regions with the largest variance among the 9 samples). **C**: Average line plot showing the DNA methylation level around the gene bodies in each cell population (TSS and TES). **D**: Numbers of DMRs identified among the 3 LM populations. **E**: CpG island annotation of DMRs identified in LSC versus LIC or LDC (CpG shore: within 2 kb from CpG island; CpG shelf: within 2–4 kb from CpG island; CpG open sea: more than 4 kb away from CpG island). Abbreviations: DMRs, differentially methylated regions; DNA, Deoxyribonucleic acid; LDC, leiomyoma differentiated cells; LIC, leiomyoma intermediate cells; LM, leiomyomas; LSC, leiomyoma stem cells; PCA, principal component analysis; TES, transcription ending site TSS, transcription starting site.

For our unbiased genome-wide evaluation of the DNA methylome and transcriptome, we performed high-throughput sequencing analyses, including MethylCap-Seq and RNA-Seq, on the 3 LM cell populations freshly sorted from patient LM tissues (Fig. 1A). MethylCap-Seq characterized the global DNA methylation landscape of each LM cell population. Leiomyoma stem cell–like cells showed a unique DNA methylome compared to LIC and LDC (Fig. 1B, n = 3 patients). On average, LSC had the highest DNA methylation level, whereas LIC had the lowest DNA methylation level (Fig. 1C). In LSC, we also identified 7020 regions with higher DNA methylation and 1842 with lower DNA methylation compared with LIC, and 8364 regions with higher DNA methylation and 1080 with lower DNA methylation compared with LDC (Fig. 1D). Leiomyoma intermediate cells and LDC had comparable DNA methylomes, with only 288 differentially methylated regions (DMRs) detected, indicating that DNA methylation plays an important role during the initial differentiation of LSC.

We evaluated the genomic distribution of DMRs between LSC and the nonstem cell populations. Compared with randomly picked genomic regions (0.7% at CpG islands and 3.5% at shores), DMRs in LSC were highly enriched in CpG islands and shores (Fig. 1E). This feature was more significant in regions with lower DNA methylation in LSC: 19.9% CpG islands and 35.7% shores were found in the LIC and LSC comparison; and 29.3% CpG islands and 47.9% shores were found in the LDC and LSC comparison (Fig. 1E). These findings were consistent with the DNA methylation pattern changes observed during intestinal stem cell differentiation using whole-genome shotgun bisulfite sequencing (13).

### Transcript levels of TET1 and TET3 methylcytosine dioxygenases are the lowest in LSC

**Figure 2.**
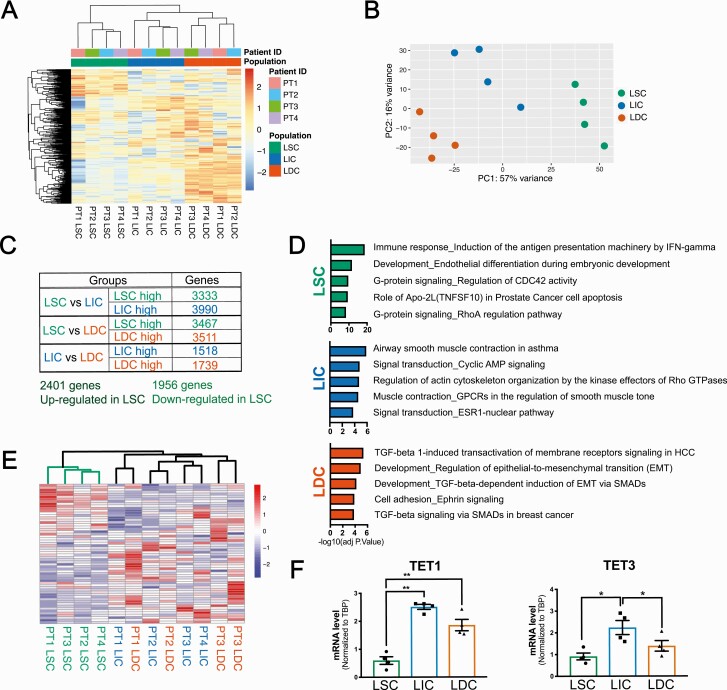
TET methylcytosine dioxygenase transcript levels are the lowest in LSC. Hierarchical clustering heatmap (**A**) and PCA plot (**B**) showing the transcriptional profiles of the 3 LM cell populations (n = 4 patients, top 1000 genes with the largest variance among the 12 samples). **C**: Numbers of DEGs identified among the 3 LM populations using RNA-Seq. A total of 2401 genes were identified with higher expression in LSC compared with either LIC or LDC; 1956 genes with lower expression in LSC compared with either LIC or LDC. **D**: Pathway enrichment analysis (Metacore) showing pathways enriched in genes that are uniquely highly expressed in each individual cell populations. **E**: Hierarchical clustering heatmap showing mRNA expression pattern of genes involved in DNA methylation and demethylation process (GSEA Gene Ontology Term [GO:M14518]) among the 12 RNA-Seq samples from the sorted LM cells (n = 4 patients). **F**: Bar graphs showing real-time qPCR validation of the differential expression of TET1 and TET3 among the 3 LM cell populations (means ± SEM, n = 4 patients, *P < 0.05, **P < 0.01, one-way ANOVA followed by pairwise comparisons). Abbreviations: ANOVA, analysis of variance; DEGs, differentially expressed genes; DNA, deoxyribonucleic acid; LDC: leiomyoma differentiated cells; LIC, leiomyoma intermediate cells; LM, leiomyomas LSC, leiomyoma stem cells; mRNA: messenger ribonucleic acid; PCA, principal component analysis; qPCR, real-time quantitative polymerase chain reaction; TET: Ten-eleven translocation.

We then performed RNA-seq to reveal possible underlying mechanisms for the distinct DNA methylation pattern in LSC. The principal component and hierarchical clustering analyses of RNA-Seq data demonstrated distinct clustering of the 12 samples (n = 4 patients) into the 3 cell populations and a matching pattern between the transcriptomes and methylomes (Fig. 2A and 2B). Consistent with previous observations based on microarray data, heatmap clustering (Fig. 2A) indicated a significant transcriptome difference between LSC and the nonstem cell populations (11). Compared to both LIC and LDC, differential gene expression analysis detected 2401 genes with higher expression and 1956 genes with lower expression in LSC (Fig. 2C). Pathway enrichment analysis demonstrated that genes highly expressed in LSC were enriched in pathways essential for stem cell maintenance and function, including immune response and G-protein signaling (34, 35), whereas genes highly expressed in LIC or LDC were enriched in pathways related to the committed cell function, including smooth muscle contraction, ESR1 nuclear pathway, and TGF-β signaling, which are known to be important for LM tumorigenesis (33, 36) (Fig. 2D).

Supporting the distinct methylation landscape identified by MethylCap-Seq, genes involved in DNA methylation and demethylation processes (Gene Ontology Term [GO:0044728]) maintained a unique expression pattern in LSC compared with the other 2 cell populations (Fig. 2E). Among the DNA methylation machineries, real-time qPCR analysis demonstrated that DNMT1 and MBD1 showed slightly higher expression in LSC, but these differences did not reach statistical significance (data not shown). Importantly, the mRNA levels of TET1 and TET3, 2 DNA demethylases, were expressed at the lowest levels in LSC (Fig. 2F), providing a possible molecular explanation for the global hypermethylation observed in the LSC population.

### DNA methylation changes correlate with gene expression changes globally during LSC differentiation

**Figure 3.**
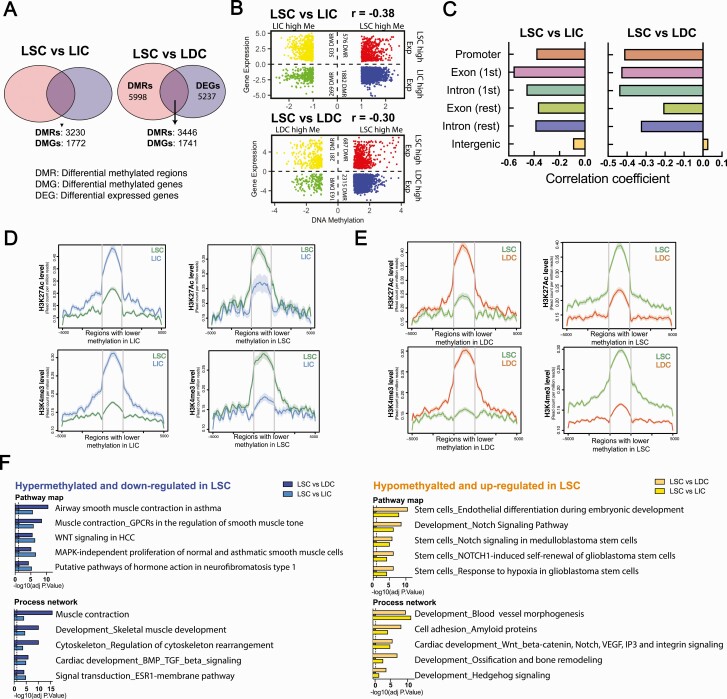
DNA methylation changes correlate with gene expression changes globally during LSC differentiation. **A**: Venn diagram showing the overlapping genes between DMGs and DEGs in the LSC versus LIC (left panel) and LSC versus LDC (right panel) comparisons. DMRs were annotated to the closest transcription start site. **B**: Dot plot showing gene expression and DNA methylation log2 fold-changes in the LSC versus LIC (top panel) and LSC versus LDC (bottom panel) comparisons. Colored dots represent regions associated with significant changes (FDR-adjusted *P *< 0.05) in both DNA methylation and expression of the nearest gene. Correlation coefficients between log2 fold-changes of gene expression and DNA methylation were calculated for both comparisons. **C**: Bar graph showing the correlation coefficient between gene expression and DNA methylation log2 fold-changes in the LSC versus LIC (left panel) and LSC versus LDC (right panel) comparisons after stratifying the genomic feature. **D**: Average line plots show the H3K27Ac and H3K4me3 enrichment level around (±5000 bp) DMRs in LSC versus LIC. **E**: Average line plots show the H3K27Ac and H3K4me3 enrichment level around (±5000 bp) DMRs in LSC versus LDC. **F**: Enrichment analysis (Metacore) showing pathways and networks highly enriched in genes that are hypermethylated/downregulated and hypomethylated/upregulated in the LSC compared with LIC or LDC. Abbreviations: DEGs, differentially expressed genes; DMGs, differentially methylated genes; DMRs, differentially methylated regions; DNA, Deoxyribonucleic acid; FDR, False Discovery Rate; LDC, leiomyoma differentiated cells; LIC, leiomyoma intermediate cells; LSC, leiomyoma stem cells.

To investigate the potential influence of DNA methylation on gene expression changes during LSC differentiation, we integrated the RNA-Seq and MethylCap-Seq data. We compared differentially methylated and expressed genes between LSC and LIC/LDC. As shown in Fig. 3A, among 8862 DMRs between LSC and LIC, 3230 regions were associated with 1772 genes differentially expressed between these 2 cell populations. Similarly, between LSC and LDC, 3446 out of 9444 DMRs were annotated to 1741 genes differentially expressed between LSC and LDC (Fig. 3A). The majority of these overlapped genes were hypermethylated, with lower expression in LSC (LSC vs LIC: 58.3% DMRs; LSC vs LDC: 67.2% DMRs; Fig. 3B, blue dots), suggesting an inverse relationship between DNA methylation and gene expression changes. Pearson correlation analysis further confirmed that DNA methylation loss during differentiation was significantly correlated with expression increase of the nearest gene (Fig. 3B; LSC vs LIC: r = -0.38, *P *< 2.2e–16; LSC vs LIC: r = -0.30, *P *< 2.2e–16; e = exponential). We found that the correlation between DNA methylation status and the expression of the nearest gene varied across the genome. For example, higher inverse correlations were found when the differentially methylated regions were near transcription start sites, including promoter, first exon, and first intron (Fig. 3C). To further investigate the possible role of DNA methylation in mediating the transcriptional activities of DNA regulatory elements, we performed ChIP-Seq for the active histone marks, H3K4me3 and H3K27Ac, in the 3 sorted cell populations (n = 3 patients). As expected, we found that during differentiation from LSC to LIC and LDC, loss of DNA methylation was strongly associated with the enrichment of H3K4me3 and H3K27Ac active histone marks (Fig. 3D and 3E).

We next performed enrichment analysis to identify pathways and networks enriched in the genes found to be both differentially methylated and expressed during LSC differentiation. We identified several processes that play crucial roles in LM tumorigenesis, including the WNT/β-catenin pathway, muscle development, BMP/TGFβ signaling, and steroid hormone-related signaling (Fig. 3F) (7, 33, 36). Notably, genes hypermethylated and downregulated in LSC (blue bars) were more enriched in pathways regulating myometrium- and LM-associated functions (eg, muscle contraction, muscle development, hormone action, and ESR1 signaling), while genes hypomethylated and upregulated (yellow bars) were involved in stem cell–related pathways.

### Genes critical for stem cell differentiation are hypermethylated and suppressed in LSC

**Figure 4.**
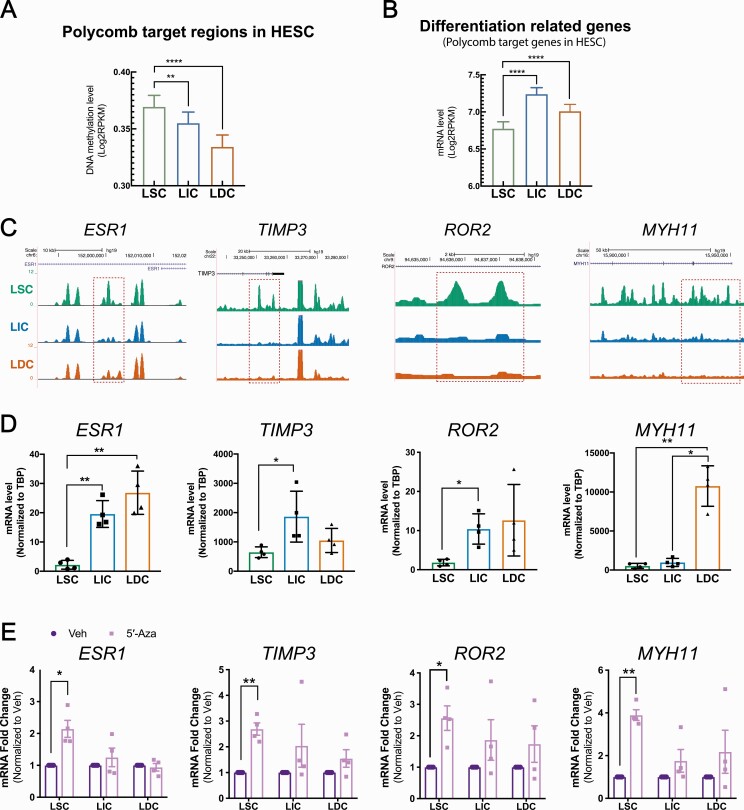
Genes critical for stem cell differentiation are hypermethylated and suppressed in LSC. Bar graph showing the average DNA methylation (**A**, region number = 2808) and mRNA (**B**, gene number = 1139) levels in LM cell populations at Polycomb target regions/genes identified from human embryonic stem cells (means ± SEM, n = 3 patients, ***P < 0.001, ****P < 0.0001, one-way ANOVA). **C**: Representative genome browser view showing DNA methylation levels at *ESR1*, *TIMP3*, *ROR2*, and *MYH11* loci (green, blue, and orange represent LSC, LIC, and LDC MethylCap-Seq, respectively). **D**: Bar graph showing mRNA levels of ESR1, TIMP3, ROR2, and MYH11 in each LM population (means ± SEM, n = 4 patients, *P < 0.05, **P < 0.01, one-way ANOVA followed by pairwise comparisons). **E**: Bar graph showing the mRNA fold-changes of ESR1, TIMP3, ROR2, and MYH11 in each LM cell population treated with 5′-Aza (96 hours, 100 nM) versus vehicle (DMSO; means ± SEM, n = 4 patients, *P < 0.05, **P < 0.01, paired t-test). Abbreviations: ANOVA, analysis of variance; DNA, deoxyribonucleic acid; LIC, leiomyoma intermediate cells; LM, leiomyoma; LSC, leiomyoma stem cells; mRNA, messenger ribonucleic acid; SE, standard deviation.

Previous studies have shown that genes governing the differentiation process are repressed by Polycomb in human embryonic stem cells (16). We found significantly higher DNA methylation and lower mRNA levels at these Polycomb targets in LSC versus LIC and LDC (16), suggesting that DNA methylation might “lock” differentiation-associated genes in a repressed state in LSC (Fig. 4A and 4B). To explore the potential role of DNA methylation during LSC differentiation, we investigated the DNA methylation-mediated regulation of several Polycomb target genes with established roles in stem cell differentiation or LM pathogenesis. Among those Polycomb targets adjacent to hypermethylated regions and inhibited in LSC, we focused on ESR1, TIMP3, ROR2, and MYH11. The Estrogen/ESR1 signaling pathway plays important roles in LM tumorigenesis (37, 38). TIMP3, tissue inhibitor of metalloproteinase 3, regulates extracellular matrix at the tumor–stromal interface (39) and is critical in driving differentiation and decreasing tumorigenesis in the mammary gland and muscle tissues (40, 41). The ROR2 pathway plays complex roles in stem cell function and carcinogenesis (42, 43), and MYH11 is a marker of fully differentiated smooth muscle cells (44).

MethylCap-Seq showed that the *ESR1*, *TIMP3*, *ROR2*, and *MYH11* gene loci were hypermethylated at several intronic regions in LSC; the *ESR1* gene was also hypermethylated at the promoter region in LSC (Fig. 4C). Opposite from the DNA methylation status, mRNA levels of ESR1, TIMP3, ROR2, and MYH11 were the lowest in LSC (Fig. 4D). To assess the effect of DNA methylation on the transcriptional activities of these genes, we treated individual cell populations with DNA methylation inhibitor 5′-Aza (100 nM) for 96 hours. 5′-Aza treatment significantly increased the mRNA levels of these genes in LSC, suggesting that the transcriptional activity of genes significant for the differentiation process were inhibited by DNA methylation in LSC (Fig. 4E).

### 5′-Aza drives LSC differentiation to reduce stemness

**Figure 5.**
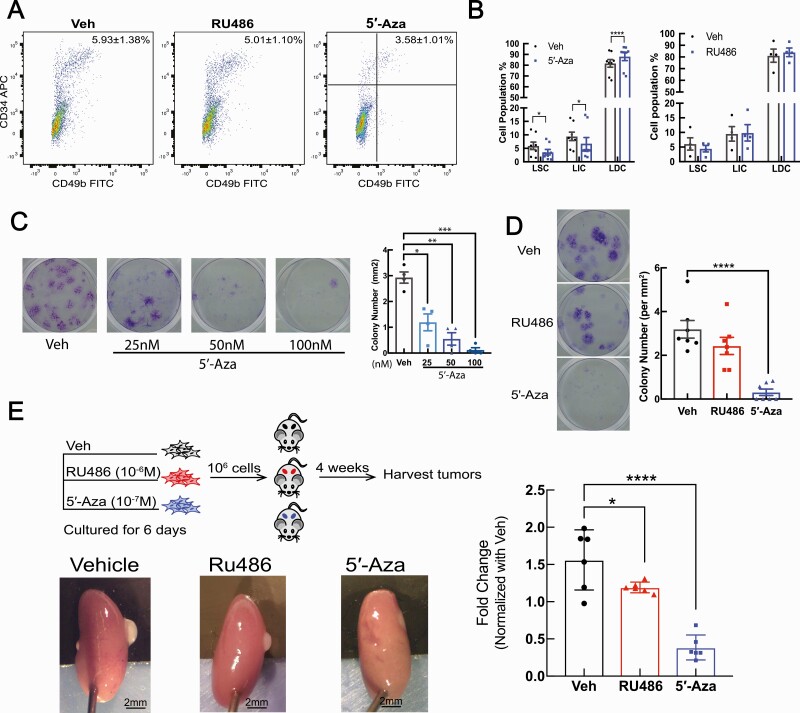
DNA methylation inhibitor 5′-Aza reduces LSC stemness. **A**: Representative flow cytometry scattergrams showing the LM cell populations isolated from LM tissue explants after a 48-hour incubation with vehicle (DMSO), 5′-Aza (100 nM), or RU486 (1 µM). **B**: Bar plots quantifying the percentage of each LM cell population in LM explants treated with vehicle, 5′-Aza (100 nM), or RU486 (1 µM; means ± SEM, 5′-Aza = 8 patients, RU486 = 4 patients, *P < 0.05, ****P < 0.0001, two-way ANOVA followed by pairwise comparisons). **C**: Colony formation assay of LM primary cells pretreated with Vehicle is DMSO (Dimethyl sulfoxide) or 5′-Aza (25 nM, 50 nM, 100 nM) for 6 days. Representative pictures of the colony formation assay (left panel); quantification of clone numbers per mm^2^ of culture area under each treatment (left panel; means ± SEM, n = 4 patients, *P < 0.05, **P < 0.01, ***P < 0.001, one-way ANOVA followed by pairwise comparisons). **D**: Colony formation assay of primary LM cells pretreated with vehicle (DMSO), 5′-Aza (100 nM), or RU486 (1 µM) for 6 days. Representative pictures of the colony formation assay (left panel); quantification of clone numbers per mm^2^ of culture area under each treatment (right panel; means ± SEM, n = 7 patients, ****P < 0.0001, one-way ANOVA followed by pairwise comparisons). **E**: Workflow of the in vivo xenograft mouse experiments using primary LM cells pretreated with vehicle (DMSO), RU486 (1 µM), or 5′-Aza (100 nM) for 6 days (top-left panel). Representative pictures of regenerated LM tumors (bottom-left panel). Quantification of the tumor volumes (right panel; means ± SEM, patient number = 3, kidney graft = 6, *P < 0.05, ****P < 0.0001, one-way ANOVA). Abbreviations: ANOVA, analysis of variance; DNA, deoxyribonucleic acid; LM, leiomyoma; LSC, leiomyoma stem cells.

We demonstrated that DNA methylation contributes to the expression changes of critical genes during LSC differentiation. We then tested the ability of 5′-Aza to regulate LSC function and compared its effect with that of RU486, a progesterone antagonist shown to inhibit LM growth (33). We treated LM tissue explants with vehicle (DMSO), RU486 (1 µM), or 5′-Aza (100 nM) for 48 hours and analyzed the proportions of each LM cell population. As shown in Fig. 5A and 5B, 5′-Aza treatment decreased around 40% of the LSC population (5.93 ± 1.38% vs 3.58 ± 1.01%). The treatment also decreased the LIC population and increased the LDC population compared to the vehicle-treated cells, whereas RU486 did not significantly change the LM cell composition. We also tested the effect of RU486 or 5′-Aza on the clonogenic activity of passage zero (unpassaged) primary LM cells, a marker of tumor stem cells (45). Cells were treated with vehicle (DMSO), RU486 (1 µM), or 5′-Aza (25 nM, 50 nM, or 100 nM) for 6 days, and 500 viable cells from each treatment group were plated in each well of a 12-well plate and cultured for 21 days without further treatment. We found that pretreatment with 5′-Aza markedly decreased colony formation in primary LM cells even at a very low dose (25 nM), whereas RU486 did not have a significant effect (Fig. 5C and 5D). In addition, we compared the tumor initiation capacity of passage zero primary LM cells (1 x 10^6^ viable cells) pretreated with vehicle, 5′-Aza, or RU486 for 6 days. Although the alteration of cell surface marker gene expression during in vitro culture hindered us from distinguishing cellular components of primary LM cells after culture, our previous studies and the current colony formation assay indicate the presence of LSC in cultured primary LM cells (7, 46). We found that primary LM cells pretreated with 5′-Aza regenerated significantly smaller tumors (36.30 ± 3.57% of vehicle size) compared with RU486-pretreated (76.31 ± 1.86% of vehicle size) or vehicle-pretreated primary LM cells (Fig. 5E).

**Figure 6.**
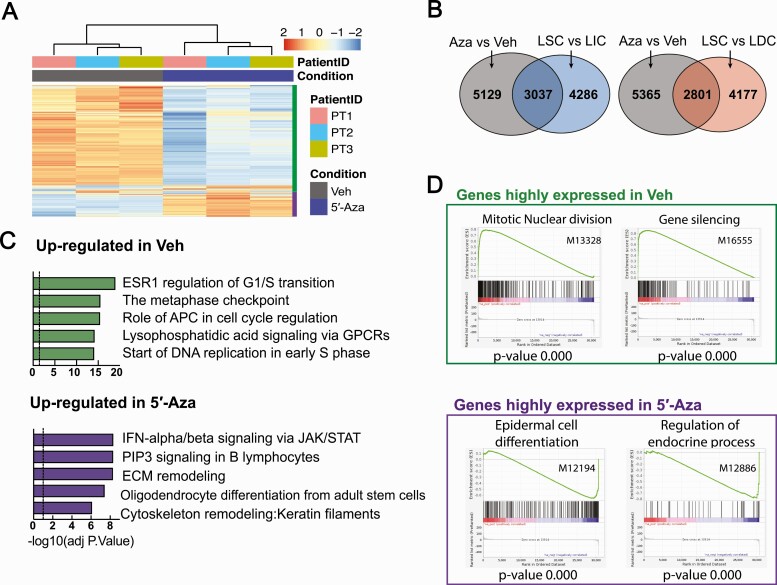
DNA methylation inhibitor 5′-Aza significantly shifted the transcriptome of LM cells. **A**: Hierarchical clustering heatmap of RNA-Seq showing the transcriptome profiles of primary LM cells treated with vehicle (DMSO) or 5′-Aza (100 nM) for 6 days. **B**: Venn diagram showing the overlap of differentially expressed genes after 5′-Aza treatment and during LSC differentiation. **C**: Top and bottom panels represent enrichment analysis (Metacore) of genes downregulated and upregulated by 5′-Aza (100 nM, 6 days), respectively. **D**: Gene enrichment analysis of RNA-Seq data in (**A**) based on C5 (GO terms) collection in the GSEA database. Top and bottom panels represent GO terms of genes downregulated and upregulated by 5′-Aza, respectively. Abbreviations: DNA, deoxyribonucleic acid; LM, leiomyoma; LSC, leiomyoma stem cells.

We further explored the molecular mechanisms underlying 5′-Aza-mediated LSC population decrease and LM growth inhibition. We performed RNA-Seq on vehicle- (DMSO) or 5′-Aza- (100 nM, 6 days) treated primary LM cells. The global transcriptome changed markedly with 5′-Aza treatment with clear clustering based on treatment status (n = 3 patients, Fig. 6A). We identified 3046 genes that were upregulated and 5120 genes that were downregulated in the 5′-Aza group compared with the vehicle group. When comparing these genes to the genes differentially expressed in LSC, we found that 5′-Aza regulated 41.5% differentially expressed genes in LSC versus LIC and 40.1% differentially expressed genes in LSC versus LDC, suggesting that DNA methylation plays a critical role in regulating transcription activities during stem cell differentiation in LM (Fig. 6B). Pathway enrichment analysis revealed that genes downregulated by 5′-Aza were related to cell cycle regulation, whereas genes upregulated by 5′-Aza were enriched in immune response activation, stem cell differentiation, and extracellular matrix remodeling pathways (Fig. 6C). Gene set enrichment analysis showed similar results, with 5′-Aza treatment significantly downregulating proliferation-related genes and upregulating differentiation- and endocrine-related genes (Fig. 6D), consistent with our in vivo findings. Together, these findings suggest that inhibition of DNA methylation decreases LM tumor regeneration ability, potentially through inducing LSC differentiation and exhaustion.

## Discussion

We demonstrated that LSC harbor a unique transcriptome and DNA methylome. Globally, hypermethylation in LSC suppresses key genes essential for stem cell differentiation, including ESR1, TIMP3, ROR2, and MYH11. The DNA methylation inhibitor 5′-Aza upregulated expression of genes necessary for differentiation, decreased the stem cell population by stimulating LSC differentiation, and reduced LM regeneration.

DNA methylation is a common mechanism for cell programming in both normal tissue growth and tumor development (47). Integrated analyses of genome-wide DNA methylation maps and expression profiles have revealed a hierarchy of cellular differentiation in hematopoietic tissue and the breast (48, 49). Transcriptomic analyses of LM populations have also strongly suggested a hierarchical differentiation order: LSC→LIC→LDC, while MethylCap-seq analysis showed a similar pattern of alterations in DNA methylation, particularly a transition from LSC to LIC or LDC. We found, however, very little difference between LIC and LDC with respect to DNA methylation, suggesting that a change in DNA methylation status is a critical epigenetic modification during the initial differentiation of LSC. We uncovered regions that had lost or gained methylation during LSC differentiation and that were overrepresented in CpG islands and shores, consistent with the DNA methylation pattern changes seen during intestinal stem cell differentiation observed using whole-genome shotgun bisulfite sequencing (Fig. 1E) (13). The functional significance of this regional preference in stem cell differentiation warrants further investigation.

DNA methylation, the most common and well-studied epigenetic mark, has a major influence on transcription activity. In our study, we found an inverse relationship between DNA methylation and gene expression changes when comparing LSC with LIC and LDC. The correlation was stronger when the methylation changes took place near the transcription start sites. It is well-known that hypermethylation at the promoter, especially a CpG dense promoter, is an indicator of suppressed transcription activity, but the role of methylation within the gene body regions remains ambiguous (50). Previously, gene body methylation was reported to be positively correlated with gene expression, but recent studies have demonstrated that methylation at the first intron and exon possesses a negative influence on the transcription activity, which coincides with our observation (51–53). Importantly, hypermethylated regions in LSC were functionally associated with transcriptional suppression of genes enriched in pathways regulating differentiated cell function, such as muscle contractions and hormone action, which is consistent with other tumor stem cell systems (Fig. 3F) (14, 15).

Consistent with previous reports showing that DNA hypomethylating agents can remove the epigenetic “brake” and allow stem cells to undergo differentiation (17), we found that the DNA methylation inhibitor 5′-Aza not only upregulated expression of genes imperative for differentiation but also functionally depleted the LSC population and inhibited its colony formation (20, 54). 5′-Aza-induced LSC differentiation was supported by the observation that 5′-Aza treatment significantly increased the expression of markers of fully differentiated smooth muscle cells, such as MYH11 (44). Notably, we found that RU486 pretreatment did not significantly affect the LM cells’ colony-forming activity and was less efficient in inhibiting LM regeneration compared with 5′-Aza treatment, suggesting that LSC deficient in steroid hormone receptors may remain quiescent, constituting a small population of cells resistant to RU486 treatment. Upon treatment cessation, the persistent stem cells potentially expand and differentiate, eventually leading to LM regrowth, which may partially explain the clinically observed frequent LM regrowth after hormone therapy, a phenomenon similar to endocrine therapy resistance in breast cancer (55). Our findings that 5′-Aza treatment-activated genes related to endocrine processes, including ESR1 (Figs. 4E and 6C), indicated that it may sensitize LSC to hormone treatment. Here, we used 5′-Aza to inhibit DNA methylation as a proof-of-concept study that DNA methylation affects LSC differentiation and stemness. As a demethylation reagent, 5′-Aza is toxic (56). Thus, more studies are needed to understand the differentiation processes of LSC to identify novel interventions, which can potentiate hormone treatments to prevent LM regrowth.

In this study, we utilized the MethylCap-Seq method to evaluate the genome-wide methylation landscape of each LM cell population. MethylCap-Seq uses MBD protein to immunoprecipitate methylated DNA fragments. Besides whole-genome bisulfite sequencing, which is the gold standard technique for methylation profiling, MethylCap-Seq has the largest coverage of the genome (57). Using this method, we identified approximately 20 000 differentially methylated regions among the 3 LM cell populations, which were widely distributed across all genomic features. However, compared with methylation microarrays (EPIC array) and reduced representation bisulfite sequencing, MethylCap-Seq has the limitation of precisely quantifying methylation levels at the single-nucleotide resolution (57). In a recent study, De Mayer et al compared results obtained by using MethylCap-seq and methylation microarrays and confirmed the consistency of the 2 methods (58).

A strength of our study is the performance of genome-wide studies in freshly sorted populations of LM cells to explore the mechanisms underlying LSC differentiation. The findings derived from patient samples undergoing minimal experimental manipulation better represent in vivo conditions, producing insights with high translational relevance. Unfortunately, technical challenges with long-term in vitro culture and propagation of LSC prohibited us from performing detailed downstream mechanistic studies specifically on stem cells. Further work is needed to optimize and establish a LSC culture system that will help us better understand the functional properties of these cells.

In summary, we found that hypermethylation maintains LSC stemness through suppression of genes critical for differentiation. This finding not only sheds mechanistic light on the molecular mechanisms that drive LSC differentiation but also suggests a treatment strategy targeting the methylation status of the LSC population to prevent the formation of new LM tumors.

## Data Availability

All high-throughput sequencing data that support the findings of this study have been deposited in the Gene Expression Omnibus (GEO) under the accession code: GSE138051. All data generated or analyzed during this study are included in this published article or in the data repositories listed in References.

## Additional Information

***Disclosure Summary:*** The authors declare no potential conflicts of interest.
